# New 3′,8′′-Linked Biflavonoids from *Selaginella uncinata* Displaying Protective Effect against Anoxia

**DOI:** 10.3390/molecules16086206

**Published:** 2011-07-25

**Authors:** Jun-Xia Zheng, Yang Zheng, Hui Zhi, Yi Dai, Nai-Li Wang, Yan-Xiong Fang, Zhi-Yun Du, Kun Zhang, Ming-Ming Li, Li-Ying Wu, Ming Fan

**Affiliations:** 1 Faculty of Chemical Engineering and Light Industry, Guangdong University of Technology, Guangzhou 510006, China; E-Mails: yangzheng1986@163.com (Y.Z.); fangyx@gdut.edu.cn (Y.-X.F.); zhiyundu@yahoo.com.cn (Z.-Y.D.); kzhang@gdut.edu.cn (K.Z.); 2 School of Chinese Materia Medica, Guangzhou University of Chinese Medicine, Guangzhou 510006, China; E-Mail: zhihui@gzhtcm.edu.cn; 3 Institute of Traditional Chinese Medicine and Natural products, Jinan University, Guangzhou 510632, China; E-Mails: tghao@jnu.edu.cn (H.G.); sy-daiyi@jnu.edu.cn (Y.D.); 4 Key Lab for Research & Development of New Drugs, Research Institute of Tsinghua University in Shenzhen, Shenzhen 518057, China; E-Mail: wangnl888@sina.com; 5 Institute of Basic Medical Sciences, Beijing 100850, China; E-Mails: ming_sunflower@sina.com (M.-M.L.); liyingwu6160@yahoo.com.cn (L.-Y.W.); fanming@bmi.ac.cn (M.F.)

**Keywords:** *Selaginella uncinata* (Desv.) Spring, 3′,8′′-linked biflavonoids, protective effect against anoxia, (2′′*S*) 2′′,3′′-dihydroamentoflavone-4′-methyl ether

## Abstract

Seven 3′,8′′-linked bioflavonoids, including one new compound, (2′′*S*)-2′′, 3′′-dihydroamentoflavone-4′-methyl ether (**1**) and six known compounds: (2*S*)-2,3- dihydroamentoflavone-4′-methyl ether (**2**), (2*S*,2′′*S*)-2,3,2′′,3′′-tetrahydroamento- flavone-4′-methyl ether (**3**), (2*S*,2′′*S*)-tetrahydroamentoflavone (**4**), (2*S*)-2,3-dihydro- amentoflavone (**5**) and (2′′*S*)-2′′,3′′-dihydroamentoflavone (**6**) and amentoflavone (**7**), were isolated from the 60% ethanolic extract of *Selaginella uncinata* (Desv.) Spring. The structures of these compounds were elucidated mainly by analysis of their 1D and 2D NMR spectroscopic data, and their absolute configurations were determined by circular-dichroism (CD) spectroscopy. All the seven compounds showed protective effect against anoxia in the anoxic PC12 cells assay, in which compound **6** displayed particularly potent activity.

## 1. Introduction

*Selaginella uncinata* (Desv.) Spring, which is a Chinese herbal medicine known as “Cui Yun Cao”, is widely distributed in the southwest China and used to treat jaundice, dysentery, edema and rheumatism diseases. [1] Earlier phytochemical investigation into this plant led to the isolation of several biflavonoids, flavonoids, chromone glycosides and phenolic constituents [2,3,4].

Hypoxia is a common environmental stress in high altitude. It can influence signaling pathways and cell functions, so several cell types including neuroendocrine chromaffin cells have evolved to sense oxygen levels and initiate specific adaptive responses to hypoxia. As a rat pheochromocytoma cell line derived from a tumor of adrenal medulla chromaffin tissue, PC12 is an oxygen-sensitive cell type and a useful system to study the effects of hypoxia [5].

**Figure 1:**
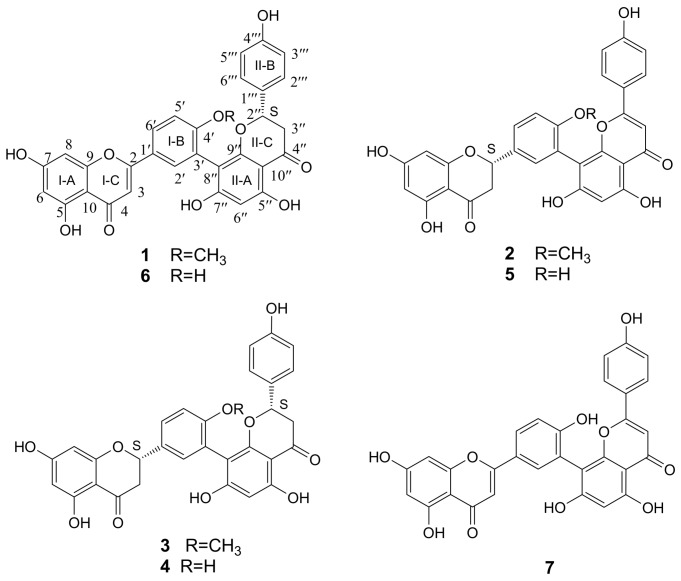
Chemical structures of compounds **1**-**7**.

In a previous search for compounds displaying protective effects against anoxia, the 60% ethanolic extract of *S. unicinata* displayed potent effects in the anoxic PC12 cells assay. Recently, we have reported 3′,6′′-linked biflavonoids isolated from the EtOAc-soluble fraction of the 60% EtOH extract of the whole plant [6]. In continuation of the research on the EtOAc-soluble fraction, seven amentoflavone-type biflavonoids have now been isolated, including one new compound (2′′*S*)-2′′,3′′-dihydroamentoflavone- 4′-methyl ether (**1**) and six known compounds **2-7** (Figure 1).

## 2. Results and Discussion

The EtOAc soluble fraction was subjected to silica gel, Sephadex LH-20 and ODS column chromatography, and finally purified by preparative reverse-phase HPLC to afford seven compounds. The seven compounds showed positive reaction with Mg/HCl, which indicated that they were flavonoids.

Compound **1** was isolated as an amorphous yellow powder. The UV spectrum of the compound was typical of biflavanones, with a maximum at 288 nm (log ε 4.92), followed by a shoulder at 327 nm (log ε 4.79) [11]. The IR spectrum showed absorption bands at 3351, 1632, 1498, 1452 cm^−1^, suggesting the presence of hydroxyl, chelated carbonyl, and aromatic ring functionalities. The positive and negative ESI-MS of **1** gave peaks at m/z 577 [M+Na]^+^ and 553 [M-H]^−^, corresponding to a molecular formula of C_31_H_22_O_10_, which was confirmed by HR-TOF-MS (found m/z 577.1099, calcd. 577.1111), consistent with the compound being a biflavonoid.

**Figure 2:**
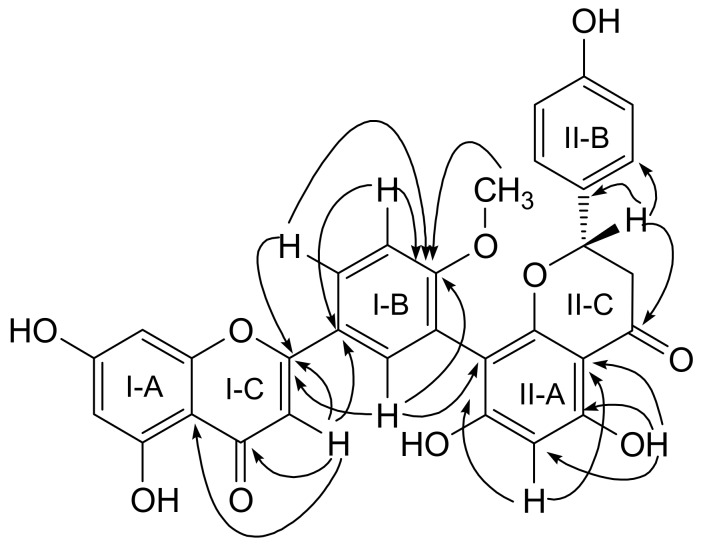
Key HMBC of compounds **1**.

In the ^1^H-NMR spectrum of **1**, one sharp singlet at *δ* 12.55 was attributed to the H-bonded OH-C (5) or OH-C (5′′), and the broad resonance at *δ* 9.51 resulted from one of the remaining nonchelated OH group (table 1). The ^1^H-NMR spectrum of **1** exhibited a one-proton singlet at *δ* 6.76 and three double doublets at *δ* 5.51 (1H, dd, *J* = 12.7, 2.9 Hz, H-2′′), 3.31 (1H, dd, *J* = 17.1, 12.7 Hz, H-3′′α), and 2.60 (1H, dd, *J* = 17.1, 2.9 Hz, H-3′′β), characteristic of a flavone and flavanone unit, respectively. The signals at *δ* 7.96 (1H, dd, *J* = 8.7, 2.2 Hz, H-6′), 7.90(1H, d, *J* = 2.2 Hz, H-2′), and 7.15(1H, d, *J* = 8.7 Hz, H-5′) revealed an AMX coupling system in the 3′, 4′-bisubstituted **I-B** ring of **1** indicating that C-3′was the position of linkage of the two flavonoid units [7]. The doublets at *δ* 7.15 (2H, d, *J* = 8.7 Hz, H-2′′′, 6′′′) and 6.60 (2H, d, *J* = 8.7 Hz, H-3′′′, 5′′′) could be assigned to the AA′XX′ spin system of the symmetric para-substituted **II-B** ring of **1**. Two *meta*-coupled proton signals at H-6 and H-8 in **I-A** ring of **1** appeared at *δ* 6.22 (1H, d, *J* = 2.0 Hz, H-6) and 6.54 (1H, d, *J* = 2.0 Hz, H-8). Further, one proton signal at H-6′′ in **II-A** ring of **1** appeared at *δ* 6.05 (1H, s, H-6′′), which was confirmed by an HMBC experiment (Figure 2). The remaining signal at *δ* 3.72 (3H, s) showed a cross-peak with *δ* 7.15 (H-5′) in the NOESY spectrum, which confirmed that the OCH_3_ group is attached to C-4′.

The ^13^C-NMR spectrum (Table 1) showed signals for 31 carbons, including two carbonyl groups (*δ* 196.2 and 181.9) and one methoxyl group (*δ* 55.6). All the chemical shifts of carbons connected with protons were confirmed using the HSQC experiment. In the HMBC spectrum (Figure 2), the OH-5′′ signal at *δ* 12.55 was correlated with resonance at *δ* 95.4 (C-6′′) and *δ* 101.1 (C-10′′); the H-2′ signal at *δ* 7.90 correlated with C-8′′ signal (*δ*104.6) and the H-6′′signal at *δ* 6.05 with resonance at C-5′′ (*δ*160.5), C-7′′ (*δ*164.2), C-8′′ (*δ*104.6) and C-10′′ (*δ*101.1), indicating that **1** was a biflavonoid with a C-3′′-C-8′′interflavonoid linkage corresponding to the amentoflavone series [8]. The ^1^H- and ^13^C-NMR signal assignments were achieved by combination of ^1^H-^1^H COSY, HSQC and HMBC spectral elucidation, and comparison with literature values of 2′′,3′′-dihydroamentoflavone [9]. On comparison of the ^13^C-NMR spectrum (Table 1) with that of 2′′,3′′-dihydroamentoflavone, it was observed that C-1′ showed a downfield shift of △1.8 ppm and C-5′ showed an upfield shift of △5.2 ppm in **1**, respectively.

The CD spectrum of 1 showed a positive Cotton effect at the n→π* absorption band of 327 nm ([Δε]327nm +6.91) and a negative Cotton effect at the π→π* absorption band of 288 nm ([Δε]288 nm −14.59), indicating compound **1** with 2′′*S* configuration possessing a conformation with P-helicity of the heterocyclic ring and having a C2′′ equatorial aryl group. Furthermore, C-2′′ was assigned the *S* configuration in accordance with literature values of compound (2′′*S*)-tetrahydroamentoflavone [10]. Thus, compound **1** is a new compound and was identified as (2′′*S*)-2′′, 3′′-dihydroamentoflavone-4′- methyl ether.

Along with the new compound, six known biflavonoids including (2*S*)-2,3-dihydroamento- flavone-4′-methyl ether (**2**) [11], (2*S*,2′′*S*)-2,3,2′′,3′′-tetrahydroamentoflavone-4′-methyl ether (**3**) [11], (2*S*,2′′*S*)-tetrahydroamentoflavone (**4**) [12], (2*S*)-2,3-dihydroamentoflavone (**5**) [13], (2′′*S*)-2′′, 3′′-dihydroamentoflavone (**6**) [9], amentoflavone (**7**) [14] were also isolated.

The protective effect against anoxia of compounds **1**-**7** was evaluated by the anoxic PC12 cells assay (Table 2). All seven compounds showed protective effect against anoxia, with compound **6** displaying the most potent protective effect, while the other compounds showed moderate effects.

## 3. Experimental

### 3.1. General

UV spectra were obtained on a Shimadzu UV2401PC spectrophotometer and IR spectra were recorded on a Shimadzu FTIR8900 spectrophotometer using KBr disks. Optical rotations were measured on a Jasco P-1020 digital polarimeter and CD spectra were recorded on a JASCO 810 spectropolarimeter. NMR data were obtained on a Bruker AV-400 spectrometer, with TMS as an internal standard. Mass spectra were determined on a Bruker Esquire 2000 spectrometer, and HR-ESI-MS were acquired using a Micromass Q-TOF mass spectrometer. HPLC analyses were carried out on an Agilent 1100 Series instrument equipped with an PDA detector, using an analytical Shim-pack VP-ODS (4.6 × 250 mm, 5 μm, Shimadzu) or a preparative Shim-pack VP-ODS (20 × 250 mm, 5 μm, Shimadzu) column. Column chromatography (CC) was conducted using silica gel (Qing Dao Hai Yang Chemical Group Co., Qing Dao, China), ODS-A120-S150 (YMC Co., Ltd, Komatsu, Japan), and Sephadex LH-20 (Amersham Biosciences, Uppsala, Sweden).

### 3.2. Plant material

Herbs of *S. unicinata* were collected in Guangxi Province, China, in August 2004, and were identified by Professor Sun Qi-shi (Shenyang Pharmaceutical University, Shenyang, China). A voucher specimen (No.Y01156SU) is deposited in the Department of Natural Products Chemistry, Shenyang Pharmaceutical University.

### 3.3. Extraction and isolation

The air-dried whole herbs (4.2 Kg) of *S. uncinata* were cut into pieces and refluxed with 60% (v/v) EtOH (126 L, 3 times). The dried extract (856 g) was dissolved in water and successively partitioned with EtOAc and water-saturated *n*-BuOH to give three parts, the EtOAc (160 g), *n*-BuOH (90.4 g) and H_2_O (600 g) soluble parts. The EtOAc soluble part was subjected to silica gel (200–300 mesh) column chromatography and eluted with a CHCl_3_-MeOH gradient system. Fifteen fractions were obtained. Fraction 7 (18 g), eluted with CHCl_3_-MeOH (95:5), was separated on a Sephadex LH-20 column (CHCl_3_-MeOH, 1:1), an ODS column (MeOH-H_2_O, 7:3), preparative reverse-phase HPLC (Shimadzu, 20 × 250 mm, MeOH- H_2_O-HAc, 60:40:0.1, flow rate 10 mL/min) to give compounds **1** (15 mg) and **2** (50 mg). Fraction 6 (8.5 g), eluted with CHCl_3_-MeOH (97: 3), was further separated again on a silica gel (200–300 mesh) column (cyclohexane-acetone, 6:4), a Sephadex LH-20 column (CHCl_3_-MeOH, 9:1), an ODS column (MeOH-H_2_O, 6:4), and then passed through preparative reverse-phase HPLC (Shimadzu, 20 × 250 mm, MeOH-H_2_O-HAc, 60:40:0.1, flow rate 10 mL/min), to give compound **3** (32 mg). Fraction 8 (19.2 g), eluted with CHCl_3_-MeOH (9:1), was separated on a Sephadex LH-20 column (CHCl_3_-MeOH, 1:1), an ODS column (MeOH-H_2_O, 7:3), preparative reverse-phase HPLC (Shimadzu, 20 × 250 mm, MeOH-H_2_O-HAc, 65:35:0.1, flow rate 10 mL/min) to give compounds **4** (15 mg) and **5** (50 mg). Fraction 9 (45 g), eluted with CHCl_3_-acetone (8:2-7:3), was separated on a Sephadex LH-20 column (CHCl_3_-MeOH, 1:1), an ODS column (MeOH-H_2_O, 7:3), preparative reverse-phase HPLC (Shimadzu, 20 × 250 mm, MeOH-H_2_O-HAc, 65:35:0.1, flow rate 10 mL/min) to give compounds **6** (17 mg) and **7** (40 mg).

*(2′′S)-2′′,3′′-Dihydroamentoflavone-4′-methyl ether* (**1**): Amorphous yellow powder; [α]${}_{D}^{27}$ +3.12° (*c* 1.0, DMSO); UV λ_max_ (MeOH) (log *ε*) nm: 327 (log *ε* 4.79), 288 (log *ε* 4.92); IRν_max_ (KBr) cm^−1^: 3351, 1632, 1498, 1452, 1343, 1254, 1165, 1087, 828; ESI-MS: *m/z* 577 [M+Na]^+^, 553 [M-H]^−^; HR-TOF-MS (positive) *m*/*z*: 577.1099 [M+Na]^+^ (calcd. for C_31_H_22_O_10_Na, 577.1111); CD (MeOH): [Δε]_327nm_ +6.91, [Δε]_288nm_ −14.59. ^1^H-NMR (DMSO-*d_6_*): Table 1; ^13^C-NMR (DMSO-*d_6_*): Table 1.

*(2S)-2,3-Dihydroamentoflavone-4′-methyl ether* (**2**): Amorphous yellow powder; [α]${}_{D}^{27}$ +6.30° (*c* 1.0, DMSO). UV λ_max_ (MeOH) (log *ε*) nm: 327 (log *ε* 5.02), 288 (log *ε* 5.20). IRν_max_ (KBr) cm^−1^: 3408, 1640, 1608, 1500, 1462, 1339, 1354, 1281, 1170, 1094, 836 cm^−1^. ESI-MS: *m/z* 577 [M+Na]^+^, 553 [M-H]^−^. HR-TOF-MS (positive) *m/z* 577.1099 [M+Na]^+^ (calcd. for C_31_H_22_O_10_ Na, 577.1111). CD (MeOH): [Δε]_327nm_ +5.76, [Δε]_288nm_ −13.87.

*(2S,2′′S)-2,3,2′′,3′′-Tetrahydroamentoflavone-4′-methyl ether* (**3**): Amorphous yellow powder; [α]${}_{D}^{27}$ −3.80° (*c* 1.0, DMSO). UV λ_max_ (MeOH) (log *ε*) nm: 289 (log *ε* 4.66), 203 (log *ε* 4.91). IRν_max_ (KBr) cm^−1^:3357, 1636, 1504, 1454, 1339, 1254, 1246, 1157, 1088, 829 cm^−1^. ESI-MS: *m/z* 579 [M+Na]^+^, 555 [M-H]^−^. HR-TOF-MS (positive): m/z 579.1240 (calcd. for C_31_H_24_O_10_Na, 579.1267). CD (MeOH): [Δε]_327nm_ +4.57, [Δε]_288nm_ −10.65.

*(2S,2′′S)-Tetrahydroamentoflavone* (**4**): Amorphous yellow powder; [α]${}_{D}^{27}$ + 2.96° (*c* 1.0, DMSO). UV λ_max_ (MeOH) (log *ε*) nm: 289 (log *ε* 4.73), 203 (log *ε* 4.95). IRν_max_ (KBr) cm^−1^: 3306, 1625, 1494, 1447, 1352, 1250, 1170, 1085, 825 cm^−1^. ESI-MS: *m/z* 543 [M+H]^+^, 541 [M-H]^−^. CD (MeOH): [Δε]_301nm_ +7.33, [Δε]_276nm_ −6.65.

*(2S)-2,3-Dihydroamentoflavone* (**5**): Amorphous yellow powder; [α]${}_{D}^{27}$ +4.48° (*c* 1.0, DMSO). UV λ_max_ (MeOH) (log *ε*) nm: 327 (log *ε* 4.31), 286 (log *ε* 4.54). IRν_max_ (KBr) cm^−1^: 3090, 1647, 1555, 1497, 1342, 1238, 1157, 1088, 833 cm^−1^. ESI-MS: *m/z* 563 [M+Na]^+^, 539 [M-H]^−^. CD (MeOH): [Δε]_327nm_ +7.24, [Δε]_286nm_ −10.45.

*(2′′S)-2′′,3′′-Dihydroamentoflavone* (**6**): Amorphous yellow powder; [α]${}_{D}^{27}$ +1.04° (*c* 1.0, DMSO). UV λ_max_ (MeOH) (log *ε*) nm: 325 (log *ε* 4.56), 289 (log *ε* 4.60). IRν_max_ (KBr) cm^−1^: 3306, 1625, 1494, 1447, 1352, 1250, 1170, 1085, 825 cm^−1^. ESI-MS: *m/z* 541 [M+H]^+^, 539 [M-H]^−^. [Δε]_325nm_ +4.06, [Δε]_289nm_ −12.33.

*Amentoflavone* (**7**): Amorphous yellow powder; UV λ_max_ (MeOH) (log *ε*) nm: 333 (log *ε* 4.79), 270 (log *ε* 4.82). IRν_max_ (KBr) cm^−1^:3082, 1651, 1609, 1574, 1493, 1423, 1358, 1285, 1242, 1165, 1107, 833 cm^−1^. ESI-MS: *m/z* 539 [M+H]^+^, 537 [M-H]^−^.

### 3.4. The anoxic PC12 cells assay

The PC12 cells were cultured in a medium that consisted of 85% DMEM (Gibco), 10% heat-inactivated (56 °C for 30 min) horse serum (Hyclone), 5% fetal bovine serum (Hyclone) and glutamine 0.10 g/L for 5 generations before used. Then, The cells were dispersed with pipette and seeded in a 35 mm culture dish at a density of 2 × 10^5^ cells/mL with 2 mL/per dish and incubated in a gas mixture of 90% air and 10% CO_2_ atmosphere at 37 °C for 8 days. The PC12 cells with samples (in 10% DMSO) were divided into groups with three dishes per group (45 μmol/L, 90 μmol/L and 180 μmol/L), respectively. A blank control group without any supplement was carried out and 10% DMSO (DMSO: normal saline = 1:9) was added to the PC12 cells as a negative control. The result showed that the toxicity of 10 % DMSO in the anoxic cells is not significant. Baicalin which displayed potent protective effect against anoxia in the PC12 cells assay was used as a positive control [15]. 24 hours after that, the cells were moved into a sealed container with 90% N_2_ and 10% CO_2_ gas for 12 hours. Cell viability was assessed by trypan blue exclusion. The viable cells were counted under inverted phase contrast microscope (400×) in 10 visual fields randomly [16]. The promoted survival rate (%) of each sample contrasted to the control was calculated. Each experiment repeats 2 times. The data was presented as mean ± SD, n = 10 visual fields and statistics were performed as Student’s T test.

## 4. Conclusions

By bioassay-guided fractionation using anti-anoxic activity, seven 3′,8′′-linked biflavonoids have been isolated from the 60% ethanol extract of the dried whole herbs of *S. unicinata*. Among these one, (2′′*S*)-2′′,3′′-dihydroamentoflavone-4′-methyl ether (**1**), is a new compound, and other six are known compounds: (2*S*)-2,3-dihydroamentoflavone-4′-methyl ether (**2**), (2S,2′′*S*)-2,3,2′′,3′′-tetra- hydroamentoflavone-4′-methyl ether (**3**), (2*S*,2′′*S*)-tetrahydroamentoflavone (**4**), (2*S*)-2,3-dihydro- amentoflavone (**5**), (2′′*S*)-2′′,3′′-dihydroamentoflavone (**6**) and amentoflavone (**7**). The protective effect against anoxia of the compounds was evaluated in the PC12 cells assay, and all seven compounds showed protective effects, with compound 6 displaying the most potent activity. The present research offers further proof that *S. unicinata* contains many bioflavonoids which display protective effect against anoxia. This finding may provide a hint for the search of new and bioactive biflavonoids from this plant.

## Figures and Tables

**Table 1:** ^1^H- and ^13^C-NMR spectral data of compound **1** (recorded at 400/100 MHz in DMSO-*d_6_*; *δ* in ppm, *J* in Hz) ^a^.

NO.	*δ*_H_ (*J*, Hz ) ^a^	*δ*c	NO.	*δ*_H_ (*J*, Hz ) ^a^	*δ*c
**2**		164.0	**2′′**	5.51 (1H, dd, *J* = 13.1, 2.8 Hz)	78.0
**3**	6.76 (1H, *s*)	102.7	**3′′**	3.31 (1H, dd, *J* = 17.1, 13.1 Hz) 2.60 (1H, dd, *J* = 17.1, 2.8 Hz)	41.2
**4**		181.9	**4′′**		196.2
**5**		161.3	**5′′**		160.5
**6**	6.22(1H, d, *J* = 2.0 Hz)	98.8	**6′′**	6.05 (1H, s)	95.4
**7**		164.1	**7′′**		164.2
**8**	6.54 (1H, d, *J* = 2.0 Hz)	94.0	**8′′**		104.6
**9**		157.3	**9′′**		162.0
**10**		103.8	**10′′**		101.1
**1′**		122.0	**1′′′**		129.6
**2′**	7.90 (1H, d, *J* = 2.2 Hz)	131.1	**2′′′/6′′′**	7.15 (2H, d, *J* = 8.7 Hz)	127.5
**3′**		122.5	**4′′′(-OH)**	9.51 (1H, br s)	157.1
**4′**		161.3	**3′′′/5′′′**	6.60 (2H, d, *J* = 8.7 Hz)	114.6
**5′**	7.15 (1H, d, *J* = 8.7 Hz)	111.2			
**6′**	7.96 (1H, dd, *J* = 8.7, 2.2 Hz)	127.3			
**OMe-4′**	3.72 (3H, s)	55.6			

^a^ br s, broad singlet; d, doublet; dd, double doublet; s, singlet.

**Table 2:** The protective effect against anoxia of compounds **1-7** in the anoxic PC12 cells assay.

Compounds	Promoted Survival Rate (%)		
	45 μmol/L	90 μmol/L	180 μmol/L
Blank control	17.81 ± 1.34		
Negative control	16.56 ±1.66	17.69 ± 1.68	15.12 ± 1.87
Baicalin	21.53 ±1.17 ^**^	26.24 ±2.08 ^**^	39.30 ± 2.31 ^**^
**1**	20.88 ±1.41 ^*^	25.92 ±2.23 ^**^	31.53 ± 2.39 ^**^
**2**	18.07 ± 1.83	23.56 ±2.64 ^**^	30.52 ± 2.49 ^**^
**3**	17.27 ±1.61	24.15 ± 1.76 ^**^	28.02 ± 2.45 ^**^
**4**	18.24 ±1.09	20.25± 1.47 ^**^	27.89 ±1.57 ^*^
**5**	16.16 ±0.21	20.88 ±1.41 ^*^	25.67 ± 1.83 ^**^
**6**	19.35 ± 1.53	24.71 ± 2.75 ^**^	38.27 ±3.24 ^**^
**7**	18.82 ± 1.23	21.07 ± 2.35 ^*^	25.76 ± 1.19 ^**^

Date were expressed as mean±SD (*n* = 3). Statistical significance was determined by Student t-test. ^*^ p < 0.05, ^**^ p < 0.01 as compared with the control.
